# More than myalgia: An unusual presentation of exertional rhabdomyolysis

**DOI:** 10.4102/safp.v63i1.5194

**Published:** 2021-01-13

**Authors:** Matthew O.A. Benedict, Anthonio O. Adefuye

**Affiliations:** 1 Department of Family Medicine, Faculty of Health Sciences, University of the Free State, Bloemfontein, South Africa; 2 Division of Health Sciences Education, Faculty of Health Sciences, University of the Free State, Bloemfontein, South Africa

**Keywords:** rhabdomyolysis, exertional rhabdomyolysis, myalgia, physical activity, skeletal muscle damage

## Abstract

Exertional or exercise-induced rhabdomyolysis (ER) is a condition in which excessive and unaccustomed physical activity results in skeletal muscle damage. The ER is a relatively uncommon condition but can have very serious consequences such as acute renal failure, severe electrolyte abnormalities, acid base disturbances and death if not recognised and managed appropriately. The risk factors for rhabdomyolysis exist in our local setting, hence, it is paramount that healthcare practitioners (GPs) in our settings be made aware of ER, its prevention and symptoms. Cases of ER are often reported in sports men or women. Here, we report a case of a 33-year-old healthy female, with clinical and serological presentation, which is typical of ER following the commencement of a regimen of exercise to lose weight.

## Background

Rhabdomyolysis is a clinical syndrome resulting from significant skeletal muscle injury and breakdown.^1^ Many cases of rhabdomyolysis are often undetected and its occurrence has been reported only in subgroups of populations at risk.^2^ Exertional rhabdomyolysis (ER) is a potentially life-threatening clinical condition that is characterised by the breakdown and necrosis of skeletal muscle induced by physical activity.^3^ Exertional or exercise-induced rhabdomyolysis is a relatively uncommon condition with an incidence of approximately 29.9 per 100 000 patient-years but can have very serious consequences such as acute renal failure, severe electrolyte abnormalities, acid-base disturbances and death if not recognised and managed appropriately.^4^ Exertional or exercise-induced rhabdomyolysis is not always evident, but early recognition of this entity and prompt intervention may prevent a serious injury or even death. Hence, healthcare professionals should be able to recognise the basic signs of ER in order to administer prompt treatment.^1^ Distinguishing features of ER include the history of intense, repetitive exercise or a sudden increase in exercise in an untrained person,^5^ associated with biochemical changes such as elevated serum creatine kinase (CK) levels five times the upper limit of normal (> 1000 IU/L) and myoglobinuria (> 1000 *μ*g/mL), which present clinically as rust-coloured urine.^6^ To make a definitive diagnosis of ER, a practitioner must combine findings from history, physical examination and serological assay.^4^ Treatment modalities include rest and hydration with intravenous (IV) fluids. Cases of ER are often reported in sports men or women.^7,8,9^ Herein, we present an unusual case of ER that occurred in a healthy individual following the commencement of a regimen of exercise to lose weight.

## Case presentation

Ms JR is a 33-year-old female who presented at the emergency department (ED) of a district hospital in the Free State province, South Africa, with 5 days’ history of generalised muscle pain. There was no history of trauma. She has been healthy, with no previous medical history of significance. No fever or any further symptom suggestive of viral or bacterial infection was observed. On her doctor’s advice, she recently commenced a regimen of exercise, because she was slightly overweight (body mass index [BMI] 27.3). Few days into her workouts, she developed muscle pains, which she was treated with ‘Mypaid’ (an analgesic containing Ibuprofen and Paracetamol), purchased over-the-counter. A few days later, she consulted her general practitioner (GP) who, according to patient report, made a diagnosis of myalgia. She was given Voltaren (Diclofenac) injection intramuscularly and was requested to continue her analgesic, take some days off work to rest and to ‘go slow’ with her workouts. There was no other history of relevance.

General examination done at the ED, revealed a young healthy looking female, not in painful distress, comfortable, although a little anxious. Her vital signs were all within normal ranges and no other significant findings were noted. Examination of the musculoskeletal system revealed mild tenderness in her quadriceps femoris and latissimus dorsi muscles with no sign of inflammation or trauma. Other systems were normal.

**FIGURE 1:**
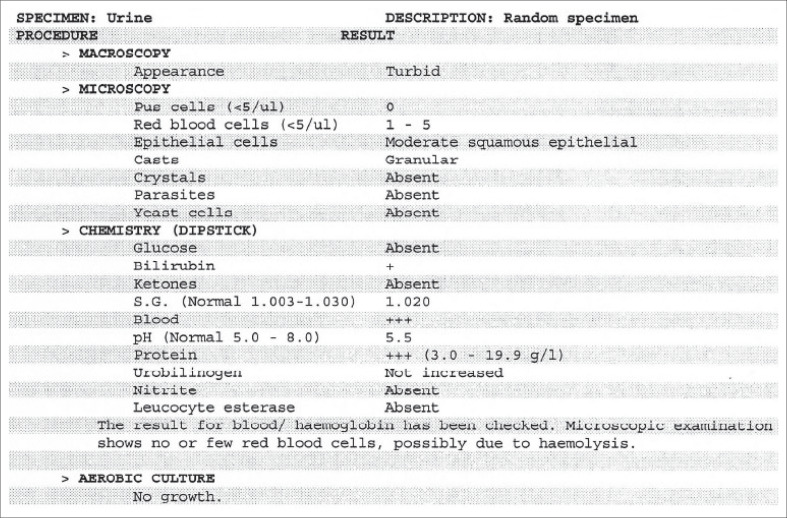
Urinalysis of the random urine sample collected at presentation at the emergency department.

Urinalysis was performed. Macroscopically, the urine was turbid and dark brown in colour, which was unusual according to the patient. She, however, thought that it was because of the analgesic she was taking. Microscopic findings include 1–5 red blood cells per microliter (reference range ≤ 5 cells/*µ*L), no pus cells, moderate squamous epithelial cells and granular casts. Crystals, parasites and yeast cells were absent. Urine chemistry (dipstick) showed blood^3+^, protein^3+^ and bilirubin^+^ (Figure 1). Other parameters were negative or normal. At this stage, a diagnosis of ER became high on the list of differential diagnoses.

Full blood count, electrolyte and urea and creatinine kinase were determined from the patient blood sample. CK was significantly elevated at 119,800 IU/L (reference range 26 IU/L–192 IU/L). Potassium level was normal (3.9 mmol/L) along with other electrolytes and urea. The full blood count was normal as well (Table 1). Based on these findings, a diagnosis of ER was made.

**TABLE 1:** Relevant investigations during admission.

Test	Reference ranges	Days on admission			
		05 October 2019 (Day 1)	06 October 2019 (Day 2)	07 October 2019 (Day 3)	08 October 2019 (Day 4)
Haemoglobin	12.1–16.3 g/dL	14.0	–	–	–
White cell	3.92–9.88	5.6	–	–	–
Sodium	136–145 mmol/L	139	137	137	–
Potassium	3.5–5.1 mmol/L	3.9	3.6	4.1	–
Chloride	98–107 mmol/L	103	104	103	–
Bicarbonate	22–29 mmol/L	23	24	24	–
Anion gap	8–20 mmol/L	17	13	14	–
Urea	< 8.4 mmol/L	3.4	3.9	3.8	–
Creatinine	49–90 μmol/L	84	71	60	–
eGFR	> 90 mL/min	79	97	115	–
CK	26–192 U/L	**119 800**	**88 787**	**83 142**	**68 948**

Note: Data in bold highlight the daily creatine kinase levels which are plotted on Figure 2.

eGFR, estimated glomerular filtration rate; CK, creatine kinase.

**FIGURE 2:**
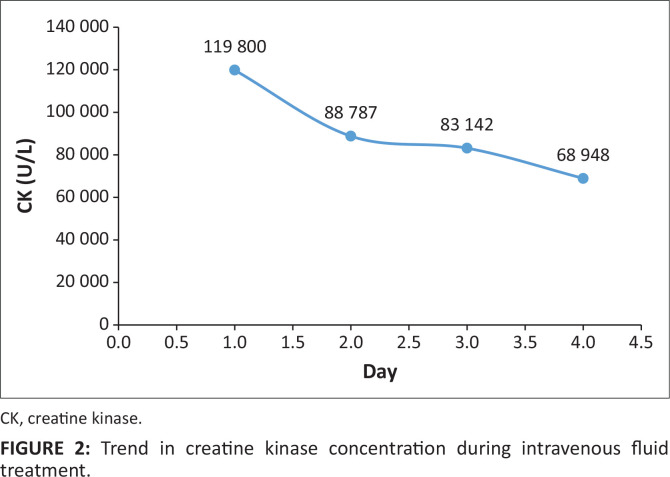
Trend in creatine kinase concentration during intravenous fluid treatment.

IV fluid (0.9% NaCl) treatment was commenced at an initial rate of 300 mL/h and the patient was admitted to a high care unit. IVfluid treatment with 0.9% NaCl was later reviewed to 500 mL/h, reaching a urinary output > 200 mL/h. During her clinical course, no complication was noted. Day 2 CK concentration was 88 787 U/L, day 3 concentration was 83 142 U/L and day 4 concentration was 68 948 U/L (Figure 2). The urea and creatinine levels remained normal and after 4 days of IV fluids, the urine was completely clear.

The patient was discharged on day 5 completely asymptomatic. No further repeats of blood tests were performed. She was subsequently booked for outpatient monitoring of her CK and renal function. The patient did not attend the follow-up clinic, however, a telephonic consultation was performed with the patient during which she indicated that she was doing well with no further complaints post discharge.

## Discussion

The causes of rhabdomyolysis are numerous and can include direct muscle injury, unaccustomed exercise, ischaemia, extreme temperatures, electrolyte abnormalities, endocrinologic conditions, genetic disorders, autoimmune disorders, infections, drugs, toxins and venoms.^10,11^ The patient in this case study was overweight, thus, fall within the population at risk of developing rhabdomyolysis was observed as described in the literature.^2^ These groups of individuals (overweight or obese) are likely to be on statins, which has been implicated in rhabdomyolysis,^12^ and are often advised to exercise more to lose weight or placed on some sort of weight-losing exercise schedule.^13^ Progressive increase in the prevalence of obesity in Africa and indeed South Africa, particularly amongst women has been reported.^14,15^ Also, trauma from inter-personal or community assault is common in our communities.^16^ Hence, there is a need for a high index of suspicion for rhabdomyolysis in our setting. In this study, the patient’s GP should have considered ER as against ‘Myalgia’ considering the recent history of unaccustomed exercise. The change in urine colour could have prompted the GP to re-consider his diagnosis, but this history was missed. It is a common practice for GPs to prescribe non-steroidal anti-inflammatory drugs (NSAIDs) for the treatment of myalgia. However, the use of NSAIDs in this scenario could ‘potentiate’ acute kidney injury and other complications. Analgesics, particularly NSAIDs, can reduce renal perfusion, leading to a depressed glomerular filtration rate.^17^

The diagnosis could have been easily missed at the ED if the attending doctor did not check the urine. The dark-brown urine was an important clue to the diagnosis, which was confirmed by the elevated CK. It is, therefore, important that in the presence of these risk factors, and an appropriate clinical setting, the urine of the patient should be properly examined and CK levels checked when history and findings on examination are suggestive of rhabdomyolysis or ER. Studies have shown several cases of non-complicated rhabdomyolysis despite CK levels over 100 000.^18^ Similarly, the patient presented herein made a complete recovery following IV hydration, despite overtly elevated CK levels. It has been reported that the outcome or prognosis in cases of rhabdomyolysis is dependent on cofactors such as the aetiology and the presence of comorbidities, and not just based on the CK levels alone.^19^ Along with IV hydration, the tracking of CK levels, kidney function and electrolyte should be monitored daily.^4^ If levels of CK continue to rise post 48 to 72 h after the presentation and depending on the severity of kidney disease or presence of compartment syndrome, consultation of a nephrologist or surgeon should be considered.^4^ The ability of medical response teams to provide aggressive hydration and dialysis services enhances survival. If treatment modalities are implemented early, patients should recover completely.^19^

The decision to discharge the patient with a CK level > 60 000 was made because the CK level was on the downward trend, urine became clear, the patient was clinically asymptomatic and other laboratory tests were normal. The acceptable discharge CK level following the treatment of rhabdomyolysis is presently debatable. Some experts have argued that discharge at higher CK thresholds of 20 000 to 50 000 U/L can be safely achieved in the ED, whilst others have recommended hospitalisation for rhabdomyolysis until CK level drops to less than 1000 U/L.^20^ Outpatient management with oral hydration may suffice for a stable patient with a CK level of 20 000 U/L to 50 000 U/L (and possibly higher), normal creatinine level and good urine flow.^21^ In a study carried out on 41 patients presenting with ER, the median discharged CK was 5287 (range, 10–61 617) U/L with a mean length of stay of approximately 3 days.^20^

## Conclusion

Patients presenting with myalgia, obese patients, patients on statins and patients taking NSAIDs are very common in our setting. Also, GPs are encouraging patients to exercise more. This suggests that the risk factors for rhabdomyolysis exist in our local setting. It is, therefore, paramount that healthcare practitioners (GPs) in our settings should be made aware of ER, its prevention and symptoms. Exertional or exercise-induced rhabdomyolysis is a relatively uncommon condition but can have severe consequences if not recognised and managed appropriately. Most resource-poor primary healthcare facilities in Africa do not have 24-h laboratory services.^22^ A high index of suspicion is, therefore, pertinent; the presence of symptoms (pain, tenderness, weakness and swelling in the muscles affected after engaging in physical activity) and typical urine discolouration are enough to prompt commencement of IV normal saline, whilst awaiting serological report on CK level.
